# Initiatives: New Voice for the Environment

**DOI:** 10.1289/ehp.115-a190a

**Published:** 2007-04

**Authors:** Nancy Bazilchuk

Intelligent design or Darwinian evolution? Life created in seven days or evolving over billions of years? Religion and science have traditionally been at loggerheads over issues like these, but in January 2007 a group of scientists and evangelicals put aside their differences to battle environmental woes such as climate change and species extinction. Under the auspices of the Harvard Medical School Center for Health and the Global Environment and the National Association of Evangelicals (NAE), scientists including biologist E.O. Wilson, climate researcher James Hansen, and botanist Peter Raven have joined forces with evangelical leaders to form a new group to educate law makers and the public about global environmental threats.

The coalition grew out of a lunchtime conversation between Eric Chivian, director of the Harvard center, and Richard Cizik, vice president for governmental affairs of the NAE, in which they realized that scientists and evangelicals shared a deep concern about the planet’s future and felt a moral obligation to act. “Whether you believe life was created in a millisecond or over three and a half billion years—that wasn’t the issue,” says Chivian. “The issue was that life on Earth is imperiled, and that we had to do something about it together.” The result was a retreat for 28 leading scientists and evangelicals in late November 2006, out of which the coalition was born.

Cizik says the NAE represents more than 45,000 U.S. churches with more than 30 million members who will now be further encouraged to work on environmental issues. Members of the coalition have already begun meeting with congressional representatives. The churches themselves are organizing various education efforts to mobilize their congregations. For example, a group of pastors are working on a packet that will be distributed to NAE member churches, which will include information such as ten things anyone can do to take care of the environment.

The coalition “makes perfect sense,” says Ben Campbell, project director for the Conservation International Faith-Based Initiative, which works cooperatively with religious groups to achieve conservation goals in countries including Columbia, Tibet, and Indonesia: “[Harvard] provides the background science and research, and [the evangelicals] present it in a context that makes sense from a religious standpoint.” As Cizik puts it, “We cannot love our neighbor if we allow the consequences of climate change, pollution, habitat destruction, species extinction, and the spread of human infection diseases to go unabated.”

The evangelical group’s decision to battle climate change has met with some internal opposition from two dozen of the movement’s more conservative Christian leaders, who wrote the NAE in early March that environmental issues would take away from the group’s other work on core moral issues, such as abortion. But at a meeting later in the month the NAE board of directors reaffirmed the group’s stance on environmental issues.

